# Fatty Liver, Hepatitus, and Cirrhosis in Sub-Human Primates Fed Ethanol

**Published:** 1995

**Authors:** Hidekazu Tsukamoto

**Affiliations:** Hidekazu Tsukamoto, D.V.M., Ph.D., is a professor of medicine and pathology, University of Southern California (USC) Center for Liver Diseases, USC School of Medicine, Los Angeles, California

**Keywords:** alcoholic liver disorder, fatty liver, liver cirrhosis, hepatitis virus, AOD effects (AODE), animal model, diet, nutritional deficit

**Figure:**
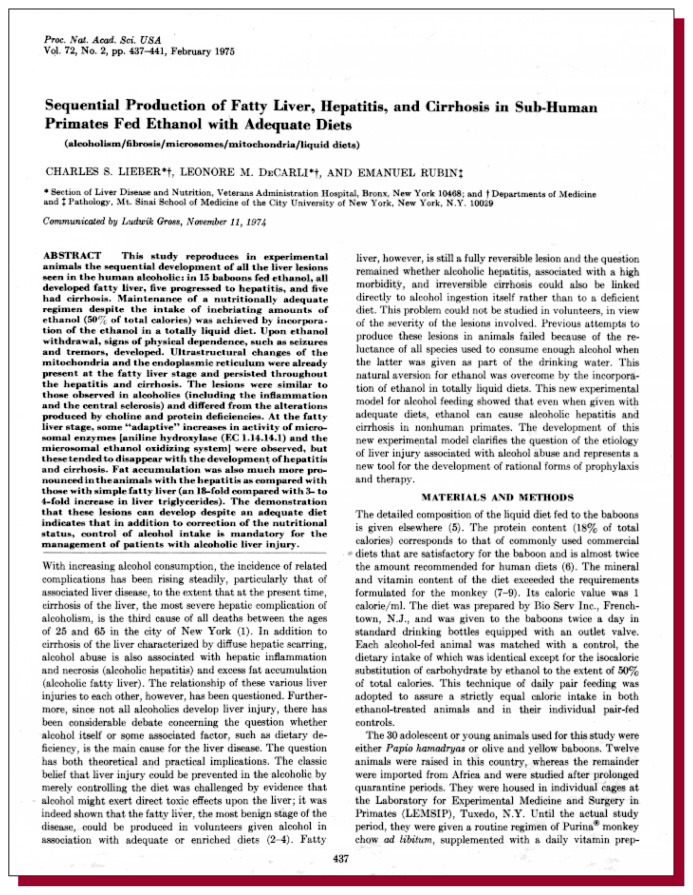
Lieber, C.S.; DeCarli, L.M.; and Rubin, E. Sequential production of fatty liver, hepatitis, and cirrhosis in sub-human primates fed ethanol with adequate diets. *Proceedings of the National Academy of Sciences USA* 72(2):437–441, 1975.

This study by Lieber and colleagues published in 1975 is one of a few seminal articles written in the past 25 years that has made a critical impact on the subsequent direction of research conducted on the mechanism of alcoholic liver disease (ALD), which usually progresses from fatty liver to hepatitis (inflamed liver with dead liver tissue) to liver fibrosis (scarring liver) to cirrhosis (severe and irreversible scarring of the liver). To comprehend fully the significance of this article, it is helpful to have a good historical perspective on the controversy that dominated the field at the time it was published. At the center of the controversy was this question: Does alcohol itself or do other factors such as dietary deficiency cause ALD? Not only was this issue scientifically important, but it also had serious practical implications.

Because patients with ALD often have accompanying malnutrition, deficiencies of key nutrients were thought to be a primary cause of this disease. This view was exemplified by the early studies of Best and colleagues (1949) and Koch and colleagues (1969), which demonstrated that alcohol-induced fatty liver could be prevented in rats when the animals’ diets were supplemented with choline or protein. Lieber and colleagues challenged this theory even though it was widely supported by the scientific community.

In their study, Lieber and colleagues fed a liquid diet containing alcohol that was originally developed for rats to a species related more closely to humans—the baboon. The researchers chose baboons because an earlier study had shown that, as a result of their aversion to alcohol, rats fed this liquid diet derived no more than 36 percent of their total calories from alcohol and failed to reproduce liver disease beyond fatty liver. Lieber and colleagues hypothesized that baboons might overcome this limiting factor and consume enough alcohol to induce more advanced ALD. Indeed, their study demonstrated that the baboons’ alcohol consumption reached 50 percent of their total calories, a percentage approximating the calorie amount that many human alcoholics derive from alcohol. In addition, these animals exhibited the sequential development of the whole spectrum of ALD, ranging from fatty liver and hepatitis to cirrhosis. Because the liquid diet was designed to provide adequate nutrition, the baboons’ development of liver disease was interpreted as evidence supporting alcohol’s causal role in the development of ALD.

Epidemiological studies of alcoholic patients by Lelbach (1975) and Pequignot and colleagues (1978) supported this conclusion, showing that the risk of developing alcoholic liver cirrhosis increases in proportion to alcohol consumption. These results implied that to manage patients with ALD successfully, alcohol intake must be controlled and that nutritional support alone might not prevent the development of the disease.

Over the past two decades, Lieber and colleagues’ findings indicating that alcohol is a primary causative agent for ALD inspired more aggressive studies on the biochemical basis for alcohol’s injurious effects on the liver. As a result, several hypotheses emerged, all of which linked the chemical breakdown of alcohol (i.e., metabolism) to the development of ALD. Alcohol metabolism generates harmful chemical products and byproducts (e.g., acetaldehyde [a metabolic product of alcohol] and free radicals [highly reactive molecules]) and creates deleterious metabolic and physiologic conditions that make the liver more susceptible to injury. Lieber continues to play a leadership role in much of this research.

Lieber and colleagues’ article described the first animal model for the spectrum of progressive and advanced ALD. The baboon model offered great potential for investigations examining the biochemistry, cell biology, and molecular biology of ALD. Popper and Lieber (1980) further clarified the progression of ALD in this animal model by meticulously examining liver specimens taken from the baboons at each stage of their disease. They revealed that evidence for the development of alcoholic hepatitis in baboons was lacking but that liver fibrosis and cirrhosis were developing directly from fatty liver. This was a provocative and significant finding for the understanding of alcoholic liver fibrosis development, because it indicated that liver fibrosis could be induced without death of liver cells and inflammation. These changes had long been considered important signals for the onset of liver fibrogenesis. This finding was confirmed by later studies showing the similar evolution of liver fibrosis in alcoholic patients.

Further research by Lieber demonstrated the following:

- Fibrosis surrounding a vein in the middle of a small anatomical unit of the liver (central vein) signals the beginning of progressive alcoholic liver fibrosis
- Transformation of the liver cells that store vitamin A (hepatic stellate cells) to the cells involved in scarring (myofibroblasts) is the cellular basis of the development of alcoholic liver fibrosis
- The role of acetaldehyde in stimulating production of fibrous proteins by hepatic stellate cells
- The effectiveness of using polyunsaturated lethicin (a type of lipid in the cell wall) as a therapy in alcoholic liver cirrhosis.

Most of these findings would not have been possible without the baboon model.

Although the baboon model has produced an enormous volume of new information concerning the mechanism of ALD, this model has not been reproduced by others (Rogers et al. 1981; Mezey et al. 1983). The possibility exists that the differences in the species (e.g., in later studies, rhesus monkeys were used as models in place of baboons) or variations in the feeding techniques might have resulted in the failure to reproduce ALD. The reproduction of the baboon model by independent investigators undoubtedly would help promote the appreciation and the wide use of the model. Emerging evidence from studies using the baboon and other animal models clearly points to how nutritional factors—both in deficiency and in excess—can sensitize the liver to the deleterious effects of alcohol and its metabolism. Use of the baboon model by different and independent laboratories may help address this issue in future research.
